# Isolation and Characterization of Integrin α9 Positive Extracellular Vesicles Derived from Human Corneoscleral Rings

**DOI:** 10.3390/life15111780

**Published:** 2025-11-20

**Authors:** Hung-Yin Lai, Ming-Chieh Hsieh, Hao-Hsiang Wu, Chien-Wei Lee, Shih-Hua Liu, Hsing-Yu Lin, Yi-Wen Chen, Chun-Chi Chiang, Yi-Ching Hsieh, Ying-Hsuen Wu, You-Ling Li, Hsiao-Fan Tung, Jennifer Hui-Chun Ho, Yi-Yu Tsai

**Affiliations:** 1 Graduate Institute of Biomedical Sciences, China Medical University, Taichung 404, Taiwan; u9801064@gmail.com (H.-Y.L.); catmingt@gmail.com (M.-C.H.); evinchen@mail.cmu.edu.tw (Y.-W.C.); 2 Department of Ophthalmology, China Medical University Hospital, China Medical University, Taichung 404, Taiwan; 010019@tool.caaumed.org.tw (C.-C.C.); 013465@tool.caaumed.org.tw (Y.-C.H.); 018146@tool.caaumed.org.tw (Y.-H.W.); 022125@tool.caaumed.org.tw (Y.-L.L.); 100677@tool.caaumed.org.tw (H.-F.T.); 3 Department of Optometry, Asia University, Taichung 413, Taiwan; 4 Translational Cell Therapy Center, China Medical University Hospital, China Medical University, Taichung 404, Taiwan; haohsiang.wu@gmail.com (H.-H.W.); icehikki@gmail.com (C.-W.L.); 5 Eye Center for Department of Ophthalmology, China Medical University Hospital, Taichung 404, Taiwan; 037642@tool.caaumed.org.tw (S.-H.L.); 100621@tool.caaumed.org.tw (H.-Y.L.); 6 Research and Development Center for x-Dimensional Extracellular Vesicles, China Medical University Hospital, Taichung 404, Taiwan; 7 Department of Bioinformatics and Medical Engineering, Asia University, Taichung 413, Taiwan

**Keywords:** extracellular vesicles, corneoscleral ring, Integrin α9

## Abstract

Corneoscleral-ring-derived extracellular vesicles represent a potential therapeutic strategy for promoting in vitro corneal wound healing. In this study, we successfully isolated and characterized extracellular vesicles from human corneolimbal tissue obtained from 42 donors, with a mean age of 51.62 ± 15.56 years. Donor-related factors such as age, corneal endothelial cell density, and underlying systemic conditions did not confound extracellular vesicle size and concentration with mean peak size of 99.52 ± 13.00 nm by nanoparticle tracking analysis. Western blotting analysis revealed positive Alix, stable expression of CD9 and CD81, and variable expression of CD63. Limbal stem cell (LSC)-associated markers, i.e., ABCG2, p63, Notch-1, and Integrin α9 were positively detected in the isolated extracellular vesicles. Notably, Integrin α9 showed stable and relatively strong expression in all samples serving a specific marker of LSC-derived extracellular vesicles. Functional assays demonstrated that LSC-derived extracellular vesicles exhibited better wound healing potency compared to extracellular vesicles derived from mesenchymal stem cells (MSCs). These findings suggest that corneoscleral-ring-derived extracellular vesicles express distinct LSC markers, including Integrin α9, and hold significant potential for application in corneal wound healing and ocular surface regeneration.

## 1. Introduction

Corneal blindness is one of the leading causes of blindness worldwide [1]. Cornea, a transparent medium, is important in providing good vision. Resting limbal stem cells (LSCs), which are enriched at the basal limbal area that separate cornea from conjunctiva and sclera, become activated and responsible for initiating division and subsequently promoting the regeneration of the corneal epithelium upon corneal injury [2]. LSCs play an essential role in maintaining homeostatic renewal and transparency of the cornea by not only differentiation and migration but also paracrine support and cell–cell communication through extracellular vesicles [3,4,5]. Previous studies revealed that maintenance of LSC markers is essential for corneal epithelial regeneration. Thus, preserving LSC function is key for corneal repair [6].

Extracellular vesicles, a 30–150 nm extracellular bi-lipid layer vesicle generated from the late endosome, regulates physiological and pathological conditions through several mechanisms, including fusion with plasma membrane for cargo release, endocytosis, or interaction with the surface proteins [7,8]. Extracellular vesicles participate in intercellular communication by transporting mRNA, DNA, microRNAs, and proteins, facilitating physiological crosstalk with recipient cells. Extracellular vesicles derived from stem cells have emerged as a focus of research, owing to their distinct biological properties and therapeutic promise. Stem-cell-derived extracellular vesicles have several advantages for clinical applications compared to stem cell transplantation, including lower immunogenicity and non-tumorigenicity [5,9,10]. In addition, constituents of extracellular vesicles derived from parental stem cells give diverse therapeutic potential for tissue repair and regeneration [11]. In recent years, clinical trials utilizing extracellular vesicles secreted by stem cells are emerging for regenerative medicine, disease diagnosis, or drug delivery and have been widely addressed [5]. Stem-cell-derived extracellular vesicle therapy is a novel therapeutic approach in tissue repair and immune modulation. Previous studies revealed that stem-cell-derived extracellular vesicles have benefits for corneal wound healing [12,13,14]. Thus, the intercellular communication feature of extracellular vesicles provides treatment of ocular disease and regeneration. More recently, proteomic profiling of limbal epithelial stem/progenitor cell-derived small extracellular vesicles revealed enrichment of proteins related to keratinocyte development, extracellular matrix organization, and niche regulation, suggesting that both LSC function and their microenvironmental signaling are fundamental for effective corneal repair [15].

Traditionally, human LSCs are plated and maintained in a feeder system [16,17,18] or in defined culture medium [19]. However, long-term culture of human LSCs is challenging for isolating extracellular vesicles [20,21]. After corneal transplantation in patients with corneal blindness, a residual corneoscleral ring after corneal transplantation is a medical waste but contains intact limbal area with abundant LSCs. Therefore, corneoscleral ring is a good source to obtain LSC-derived extracellular vesicles without traditional procedure and long-term maintenance for LSC isolation.

The objective of this study is to identify and characterize LSC-derived extracellular vesicles from donor corneoscleral rings. In this study, we explore donor-related variations in extracellular vesicles’ characteristics, the expression of LSC-derived extracellular vesicles markers, and their potential therapeutic applications.

## 2. Materials and Methods

### 2.1. Extraction of Limbal Tissue-Derived Extracellular Vesicles

This study was approved by the Institutional Review Board of China Medical University & Hospital, Taichung, Taiwan (Registration Number: CMUH112-REC2-143) and adhered to the tenets of the Declaration of Helsinki. The corneoscleral ring, post-corneal-transplantation, was collected and prepared in the operation room, in which the iris tissue and corneal endothelium were removed with 15# blade and preserved in Optisol-GS in 4 °C, the most widely used pharmaceutical composition to preserve corneas for transplantation [22]. In this study, the corneoscleral ring fragments used in this study were centered on the cornea and extended approximately 4 mm beyond the corneal margin (limbus). The diameter of the human cornea is typically about 11.5–12.5 mm. Each corneoscleral ring was washed with phosphate-buffered saline (PBS) and cut into 6 fragments with forceps and scissors. These fragments were cultured in 6-well plates with serum-free minimum essential medium α (MEM α) for 24 h. To remove cellular debris, the differential ultracentrifugation (dUC) was performed with spin at 300× *g* for 10 min, 1200× *g* for 20 min and 10,000× *g* for 30 min. The extracellular vesicles solution was filtered with 0.22 μm membrane before identification.

### 2.2. Extracellular Vesicles Characterization

The extracellular vesicles morphology was identified by transmission electron microscopy (TEM). The particle size and size distribution were analyzed by nanoparticle analyzer. The expression of extracellular vesicle markers was also determined.

### 2.3. Transmission Electron Microscopy (TEM)

Isolated extracellular vesicles (1 μg) were resuspended in ddH2O, absorbed on carbon-form var 300 mesh grids for 30 min, fixed with 2% glutaraldehyde, and stained with 2% uranyl acetate (UA). The grids were dragged on a piece of filter paper to remove the excess of UA, allowed to dry, and examined on a JEOL 100CX electron microscope (Tokyo, Japan) at 60 kV.

### 2.4. Nanoparticle Tracking Analysis (NTA)

Suspend extracellular vesicles in Dulbecco’s phosphate-buffered saline (DPBS), then use a ZetaView^®^ PMX-120 (Particle Metrix GmbH, Inning am Ammersee, Germany) instrument equipped with dynamic light scattering (DLS) to analysis particle size. Post-acquisition settings are based on manufacturer’s recommendations. Nanoparticle tracking analysis (NTA) records videos containing the Brownian motion of each particle. The hydrodynamic diameter of the particles is determined by their diffusion behavior in the surrounding liquid. The concentration and peak size of extracellular vesicles were recorded. The polydispersity index (PDI) was calculated as the square of the number-weighted standard deviation divided by the mean, and the span was calculated as (D90 − D10)/D50.

### 2.5. Western Blotting

The extracellular vesicles were characterized for extracellular vesicles markers: CD9, CD63, CD81, and Alix; LSC markers: ATP-binding cassette subfamily G member 2 (ABCG2), p63, Notch-1, and Integrin α9. The protein concentration was quantified by using the PierceTM BCA Protein Assay kit (Thermo Fisher Scientific, Waltham, MA, USA; 23225). Then, the samples were mixed with loading buffer and denatured for 10 min at 95 °C. Samples were separated by 10% SDS-polyacrylamide gel electrophoresis and transferred onto PVDF transfer membrane (PerkinElmer, NEF1002001PK, Waltham, MA, USA). After blocking for 1 h in 5% BSA buffer, primary antibodies (CD9, Cell Signalling, #13403, 1:1000, Danvers, MA, USA; CD63, Novus, MX-49.129.5, 1:1000, Centennial, CO, USA; CD81, Cell Signalling, #52892, 1:1000; Alix, Cell Signalling, #92880, 1:1000; ABCG2, Thermo, 13-8888-82, 1:500; P63-α, Cell Signalling, #13109, 1:1000; Notch-1, Invitrogen, # MA5-32080, 1:1000, Waltham, MA, USA; Integrin alpha 9, Abcam, ab140599, 1:1000, Cambridge, UK) was added for overnight incubation at 4 °C. HRP-secondary antibody was added for 1 h at room temperature, and ECL substrate (A38556, Thermo Fisher Scientific) was added for chemiluminescence.

### 2.6. Wound Healing Test with HCE-T Cell Lines

HCE cells were seeding on Culture-Inserts 2 well. The insertion was removed after culture for 24 h. Then, eyedrops containing 2 × 10^8^ extracellular vesicles and PBS were added. The efficacy of wound healing was evaluated at 0, 2, 5, 8, 24 h. HCET cells were purchased from the RIKEN BioResource Research Center (Kyoto, Japan).

### 2.7. Nanoparticle Flow Cytometry (NanoFCM)

NanoFCM was used to identify surface markers on individual extracellular vesicles, in which fluorescent labeling was performed using dyes or antibodies targeting specific markers. After dilution to avoid particle coincidence, the cytometer was calibrated with reference beads. The samples were introduced into the instrument, where particles pass through a laser beam, and, with their light scattering, fluorescence signals were detected. The results provided information on particle size, concentration, and fluorescence intensity. We used NanoFCM to identify single extracellular vesicles expressing Integrin α9 and extracellular vesicles surface markers such as CD9, CD63 and CD81. The NanoFCM was performed by Reliance Biosciences (Navi Mumbai, India).

### 2.8. Statistical Analysis

All statistical analyses were performed using Microsoft Excel 2016, Python 3.13, and free online statistical software (Social Science Statistics, https://www.socscistatistics.com/). Continuous variables were analyzed using the Mann–Whitney U test and categorical variables were analyzed using the chi-square test or Fisher’s exact test. Spearman correlation was used to examine the relationships between extracellular vesicle properties and donor characteristics. A *p*-value of <0.05 was considered statistically significant.

## 3. Results

### 3.1. Baseline Information of Cornea Donors

A total of 42 human corneoscleral rings were obtained from corneal donors in Taiwan (*n* = 21) and the United States (*n* = 21). The mean donor age is 51.6 ± 15.6 years. Among the donors, 22 donors are male, 7 donors are female, and the sex of 13 donors is unspecified. Corneal endothelial cell density (ECD) is a key parameter influencing the success and long-term viability of corneal transplantation. The average of donor ECD is 2808.12 ± 304.20 cells/mm^2^, which is sufficient to maintain corneal transparency and ensure graft health. After corneal transplantation, the residual corneoscleral rings from these donors were immersed in Optisol-GS, transported on ice, and stored at 4 °C before extracellular vesicles isolation. The average duration from donor death to extracellular vesicle extraction was 16.88 ± 2.15 days (Table 1).

### 3.2. The ECD and Extracellular Vesicle Extraction Duration in Donors from Taiwan and the USA

**Figure 1:**
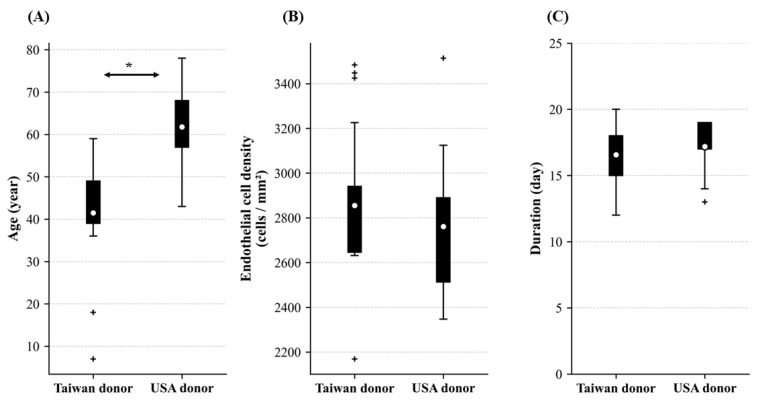
Comparison of donor characteristics between Taiwan and USA. Comparison of donor characteristics, including (**A**) age (years), (**B**) corneal endothelial cell density (ECD, cells/mm^2^), and (**C**) the duration from death to extracellular vesicle extraction (days), between donors from Taiwan and the USA. Box plots show median, quartiles, and 1.5 × IQR whiskers; + = outliers; ○ = means. *p*-values from Mann–Whitney U test. * *p* < 0.05.

In the 42 human corneoscleral rings, the Taiwan donors were younger than the USA donors (Figure 1A). The ECD of donors from Taiwan and the USA did not show a significant difference (Figure 1B). In the established protocol to isolate extracellular vesicles from human corneoscleral rings, the extracellular vesicles extraction duration was 12–20 days from donor death, and no significant difference was found in the duration of extracellular vesicles extraction between Taiwan and USA donors (Figure 1C).

When divided into subgroups by age among the 42 donors, no significant difference was found in corneal endothelial cell density, extracellular vesicle extraction duration, concentration and peak size between donor age ≧ 55 years old and <55 years old (Figure S1A–D).

### 3.3. The High Yield of Extracellular Vesicles Derived from the Corneoscleral Rings

**Figure 2:**
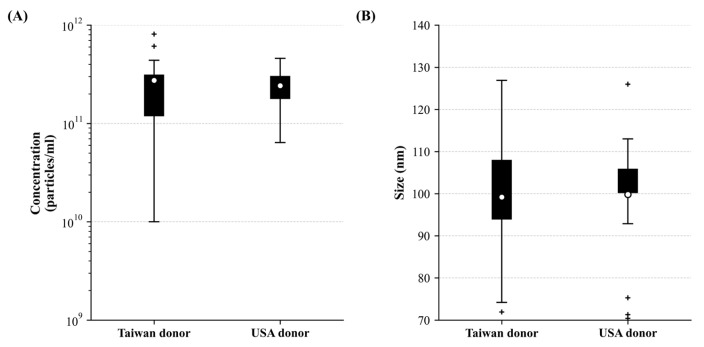
Comparison of extracellular vesicle characteristics between Taiwan and USA donor populations. Extracellular vesicle characteristics by donor origin. (**A**) Concentration (particles/mL, log scale) and (**B**) size (nm) distributions for Taiwan and USA donors (*n* = 21 each). Box plots display median, 25th–75th percentiles, 1.5× interquartile range whiskers, outliers (+), and means (○).

**Figure 3:**
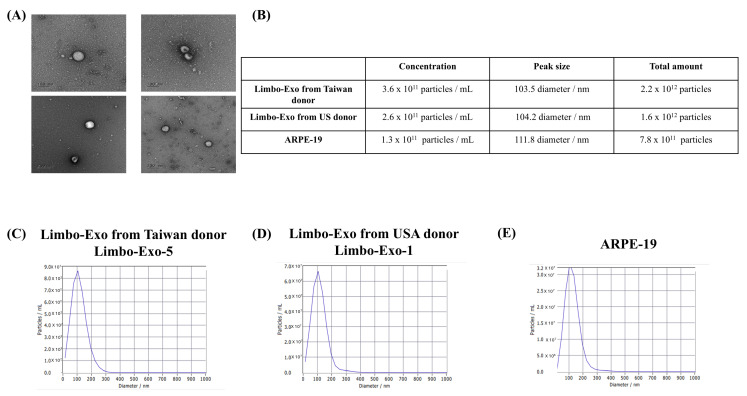
Characterization of extracellular vesicles from different sources. (**A**) Morphological analysis by transmission electron microscopy (TEM) after 24 h culture. (**B**) Comparative analysis of extracellular vesicles concentration, peak size, and total amount across different preparations. (**C**–**E**) Nanoparticle tracking analysis (NTA) of extracellular vesicle concentration and size distribution from Taiwan donors, USA donors, and ARPE-19 retinal pigment epithelial cells. RPE = retinal pigment epithelia.

To quantify and characterize the corneoscleral-ring-derived extracellular vesicles, NTA was used to analyze the concentration and peak size. The mean concentration of corneoscleral-ring-derived extracellular vesicles from Taiwan donors was 2.75 × 10^11^ particles/mL, while that from USA donors was 2.42 × 10^11^ particles/mL. The mean peak size of extracellular vesicles was 99.21 ± 13.21 nm for Taiwan donors and 99.83 ± 13.11 nm for USA donors. There was no significant difference in extracellular vesicle size and concentration between the two donor origins (Figure 2). By using TEM, the extracellular vesicles derived from corneoscleral rings revealed a typical cup shape appearance and showed a size range between 50 and 100 nm (Figure 3A). To further investigate the size distribution of corneoscleral-ring-derived extracellular vesicles, we compared the corneoscleral-ring-derived extracellular vesicles from Taiwan and USA donors (Limbo-Exo-5 and Limbo-Exo-1, respectively, from Taiwan and USA origin) with those derived from human retinal pigment epithelial (ARPE-19) cells, and found them to be similar (Figure 3C–E). Notably, the concentration and total amount of corneoscleral-ring-derived extracellular vesicles was significantly higher than in the 2-D culture of APPE-19 cells (Figure 3B). The ARPE-19 cell line was obtained from the American Type Culture Collection (ATCC). These results indicated that human corneoscleral-ring-derived extracellular vesicles exhibited a similar peak size and size distribution to those from human RPE cells, but were present in higher amounts after extraction.

### 3.4. The Correlation and Coefficient of Donor Variables on Extracellular Vesicles Size/Concentration

**Figure 4:**
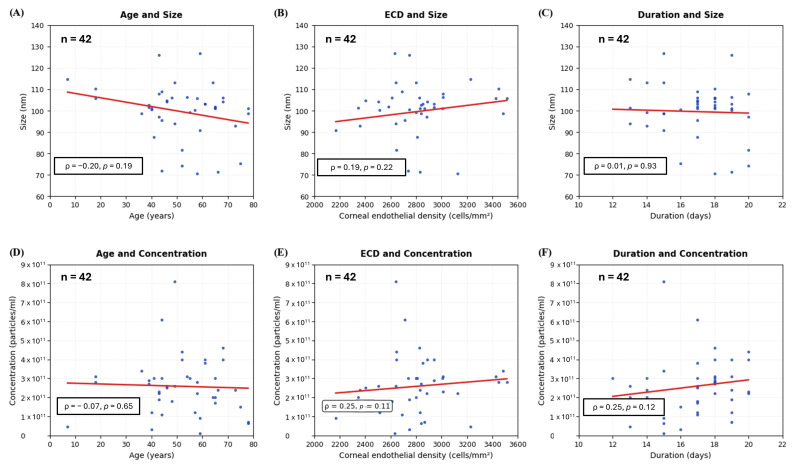
Extracellular vesicle properties versus donor characteristics. (**A**,**D**) Age correlations with extracellular vesicle size and concentration. (**B**,**E**) Corneal endothelial cell density (ECD) correlations with extracellular vesicle size and concentration. (**C**,**F**) Duration from death to extraction correlations with extracellular vesicle size and concentration. Each point represents one donor sample. Linear regression lines (red) and Spearman correlation statistics (ρ, *p*-value) are shown. All correlations were non-significant. Each scatter plot represents data from 42 donors (*n* = 42).

To investigate whether the donor variables (age, ECD, and extracellular vesicles extraction duration) could affect the peak size and concentration of corneoscleral-ring-derived extracellular vesicles, the statistical analyses were performed using Python 3.13 with the Spearman correlation coefficient. In Figure 4, the donor age did not affect the extracellular vesicle peak size and concentration. Also, the donor ECD and extracellular vesicles extraction duration did not show a discernible relationship on the extracellular vesicles peak size and concentration. Among the 42 donors, 13 donors had a history of cancer, and 3 donors had an immunodeficient condition. Separately, we conducted further analysis to investigate whether the donors’ cancer history and immunodeficient condition influence the extracellular vesicle size and extracellular vesicle concentration. As a result, there was no difference in the extracellular vesicle peak size and concentration between donors with and without cancer history (Figure S2). In addition, the donors with an immunodeficient condition did not influence the extracellular vesicle peak size and concentration compared to those from donors without immunodeficiency (Figure S2).

### 3.5. Heterogeneity of Tetraspanin Expression on Corneoscleral-Ring-Derived Extracellular Vesicles from Taiwan and USA Donors

**Figure 5:**
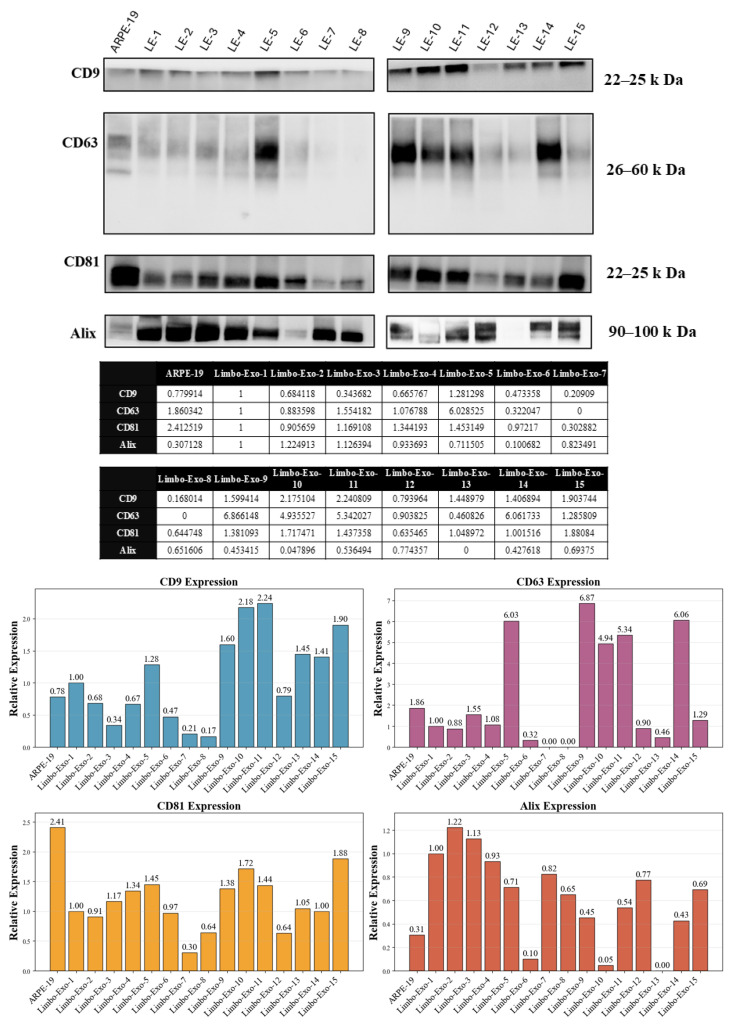
Western blot analysis of extracellular vesicle-specific markers. Representative immunoblots showing the expression of extracellular vesicle surface markers CD9 (22–25 kDa), CD63 (26–60 kDa), CD81 (22–25 kDa), and the cytosolic marker Alix (90–100 kDa) across different sample preparations (lanes 1–16). Quantitative analysis demonstrates consistent expression of these established extracellular vesicle markers, confirming successful isolation and high purity of extracellular vesicle preparations. Numerical values in the tables represent relative expression levels normalized to reference samples. LE = Limbo-Exo, RPE = retinal pigment epithelia.

Tetraspanins, including CD9, CD63, and CD81, are transmembrane proteins commonly used as markers of extracellular vesicles. Heterogeneous tetraspanin expression on extracellular vesicles was observed depending on their cellular origin [23,24]. To investigate the heterogeneity expression of corneoscleral-ring-derived extracellular vesicles, the expression of CD9, CD63, and CD81 were further analyzed (Figure 5). In the results, CD9 and CD63 exhibited high heterogeneous expression on the corneoscleral-ring-derived extracellular vesicles compared to CD81. Alix, ESCRT-associated proteins in formation of multivesicular bodies (MVBs), is also used as an extracellular vesicle marker for the origin of cellular endosomes. In Figure 5, the Alix expression was detected in the corneoscleral-ring-derived extracellular vesicles.

### 3.6. The Corneoscleral-Ring-Derived Extracellular Vesicles Presented Expression of LSC Markers

**Figure 6:**
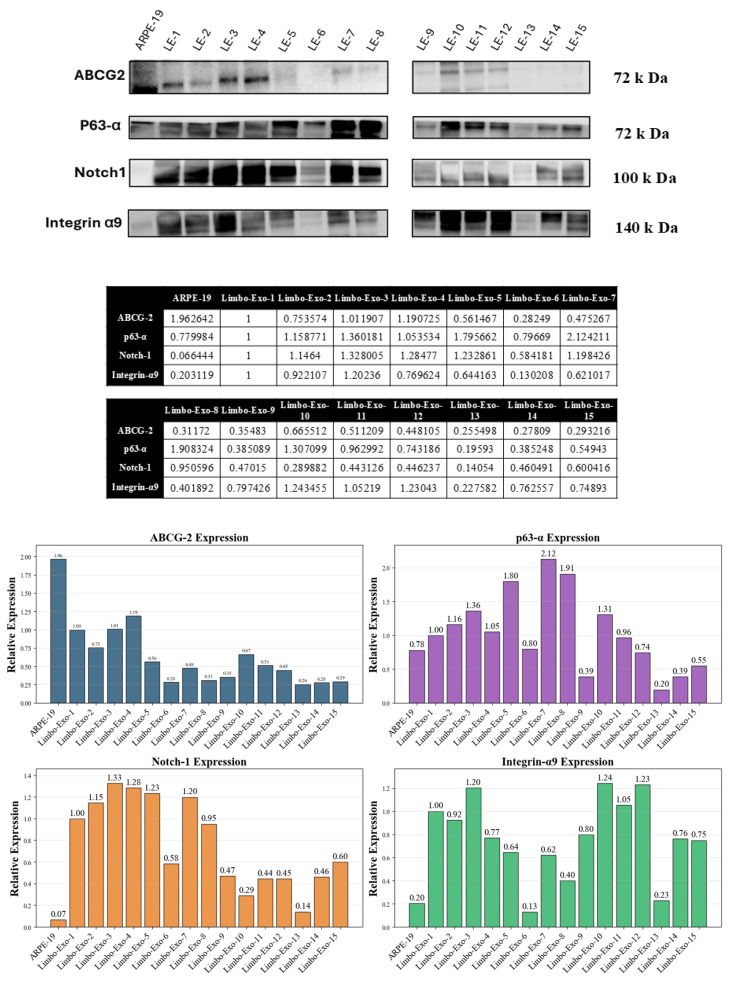
Western blot analysis of limbal stem-cell-associated markers in isolated extracellular vesicles. Representative immunoblots demonstrate the expression of key limbal stem cell markers including ABCG-2 (72 kDa), p63-α (72 kDa), Notch-1 (100 kDa), and Integrin α9 (140 kDa) in extracellular vesicles derived from different donors (Limbo-Exo-1 through Limbo-Exo-15) compared to ARPE-19 control cells. Quantitative analysis (right panels) shows relative protein expression levels normalized to Limbo-Exo-1. The presence of these established limbal stem cell markers in the isolated extracellular vesicles confirms the limbal stem cell origin and validates the successful enrichment of limbal stem-cell-derived extracellular vesicles. LE = Limbo-Exo, RPE = retinal pigment epithelia.

Cultured LSCs expressing ABCG2, p63, Notch-1, and Integrin α9 are recognized as markers distinguishing LSCs from differentiated corneal cells [25,26,27,28,29]. However, the specific markers of LSC-derived extracellular vesicles have not yet been clearly defined. To identify extracellular vesicles originating from LSCs, these common LSC markers were examined (Figure 6). The expression of ABCG2, p63, Notch-1, and Integrin α9 was analyzed and quantified in the extracellular vesicles derived from corneoscleral ring. Among them, Integrin α9 showed a more stable and constant expression compared to ABCG2, p63, and Notch-1, suggesting that it may serve as a more reliable marker for LSC extracellular vesicle markers (Figure 6).

**Figure 7:**
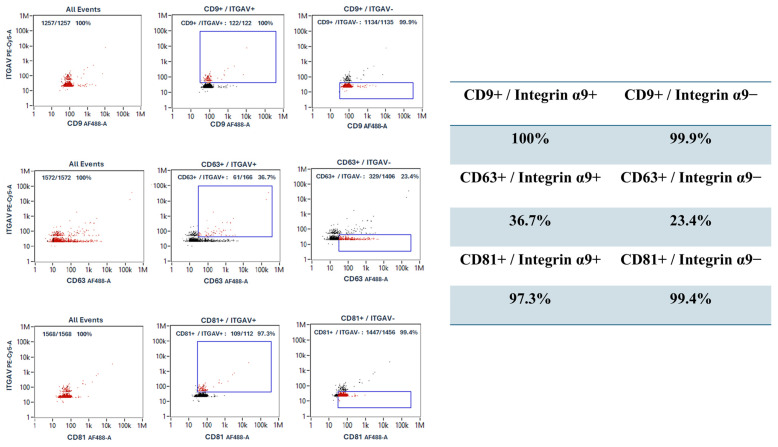
Nanoparticle flow cytometry (NanoFCM) analysis. Nanoparticle flow cytometry (NanoFCM) analysis identified extracellular vesicles subpopulations positive for integrin α9 and the tetraspanin exosomal markers CD9, CD63, and CD81. Fluorescent antibody staining enabled detection and sorting. NanoFCM experiments were performed by Reliance Biosciences.

To double-confirm corneoscleral-ring-derived extracellular vesicles with CD9, CD63, and CD81 expressions including extracellular vesicles from limbal stem cell (Integrin α9+ extracellular vesicles), nanoparticle flow cytometry (NanoFCM) (Figure 7) was performed on one sample and served as a method providing additional data, supporting the findings from Western blot analysis. The Integrin α9+ extracellular vesicles was 7–10% in the corneoscleral-ring-derived extracellular vesicles, and they showed over 95% of CD9 and CD81, while showing 36.7% of CD63. In addition, the Integrin α9− extracellular vesicles showed over 95% of CD9 and CD81, while showing 23.4% of CD63. These results indicated that Integrin α9, a limbal stem marker, is presented in approximately 7–10% of corneoscleral-ring-derived extracellular vesicles, suggesting that this subset originated from limbal stem cells.

### 3.7. Wound Healing Promotion by Corneoscleral-Ring-Derived Extracellular Vesicles

**Figure 8:**
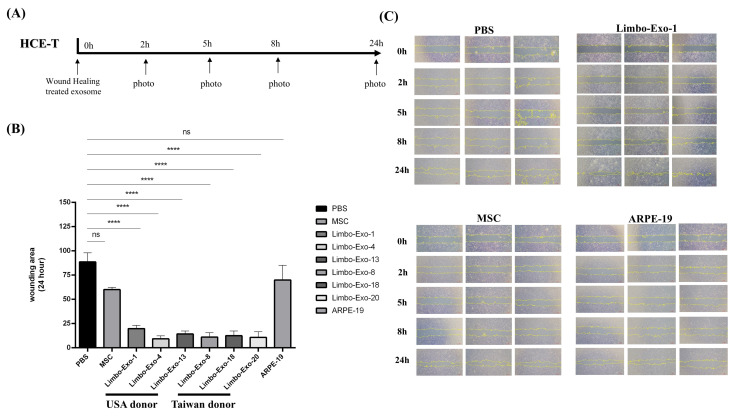
HCE-T cell wound healing assay. (**A**) The process of wound healing assay. (**B**) Wound area of HCE-T cell cultures after 24 h with extracellular vesicles derived from corneoscleral rings and the control. (**C**) Representative images from in vitro wound healing assay of HCE-T cell cultures treated with extracellular vesicles from corneoscleral rings that performed cell invasion into the cell-free region (outlined) are accelerated in the extracellular vesicles from corneoscleral rings compared to the control. Control = PBS, extracellular vesicles from MSC, extracellular vesicles from ARPE-19. HCE-T = Human Corneal Epithelial–Transformed cell, PBS = phosphate-buffered saline, MSC = mesenchymal stem cells, RPE = retinal pigment epithelia. **** *p* < 0.0001.

To investigate the therapeutic efficacy of extracellular vesicles for ocular surface repair, an in vitro wound healing assay was performed using HCE-T cells. Corneal wound healing ability of the corneoscleral-ring-derived extracellular vesicles was evaluated and compared with that of extracellular vesicles derived from mesenchymal stem cells (MSCs) and RPE cells. At the 24 h follow-up, corneoscleral-ring-derived extracellular vesicles collected from six randomly selected different donors exhibited significantly superior wound healing properties compared with extracellular vesicles from MSCs and RPE cells (Figure 8).

**Figure 9:**
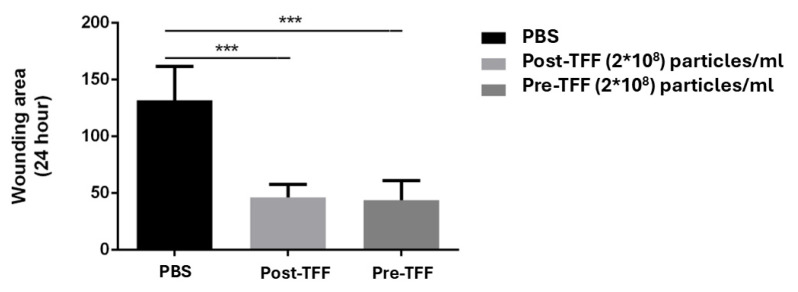
HCE-T cell wound healing assay between extracellular vesicles derived from corneoscleral rings processed with or without tangential flow filtration (TFF). Comparison of 24 h wounding area among PBS, Post-TFF (2 × 10^8^ particles/mL), and Pre-TFF (2 × 10^8^ particles/mL) treatments. Data represent mean ± SD. Statistical significance is indicated by *** (*p* < 0.001). Both Post-TFF and Pre-TFF treatments significantly reduced the wounding area compared to PBS control. There was no significant difference between Post-TFF and Pre-TFF treatments. PBS: phosphate-buffered saline; TFF: tangential flow filtration.

To confirm that the wound healing ability was attributable to extracellular vesicles derived from corneoscleral ring, the remaining soluble proteins were eliminated by tangential flow filtration (TFF) [30]. At an identical concentration of 2 × 10^8^ particles/mL, no significant difference in the wound healing efficacy was observed between the extracellular vesicles processed with and without TFF (Figure 9). The results indicated that the wound healing potency is intrinsically mediated by the extracellular vesicles, rather than by soluble proteins or RNA present in the surrounding solution.

## 4. Discussion

In this study, we successfully identified and characterized extracellular vesicles derived from human corneoscleral rings, highlighting the valuable impact of understanding LSC-derived extracellular vesicles and their unique biological properties. Through comprehensive molecular investigations, we identified, for the first time, Integrin α9 as a distinctive marker of LSC-derived extracellular vesicles, revealing key molecular markers behind their origin and function. This discovery establishes a potential biomarker for tracking and distinguishing these extracellular vesicles. Moreover, our comparative analysis revealed that extracellular vesicles derived from corneoscleral rings exhibit better wound healing potency compared to extracellular vesicles derived from MSCs. These findings suggest that corneoscleral-ring-derived extracellular vesicles may offer considerable therapeutic benefits, particularly for ocular surface repair.

Extracellular vesicles, secreted by all cell types, have been used to treat Sjögren’s syndrome dry eye, corneal allograft rejection, autoimmune uveitis, and age-related macular degeneration (AMD) [31]. Compared to cell therapy, extracellular vesicles are cell-free and offer benefits such as high cell permeability, low immunogenicity, and low tumorigenicity [32]. Physiologically, LSCs are responsible for the renewal of the corneal epithelium [33]. In the current study, we report a procedure for collecting a large number of extracellular vesicles from corneoscleral rings within 24 h which contain LSC-derived extracellular vesicles. Specifically, among common LSC markers, i.e., ABCG2, p63, Notch-1, and Integrin α9, Integrin α9 is strongly expressed in most of the donors, serving as a marker for LSC-derived extracellular vesicle identification and purification.

Extracellular vesicles derived from retinal pigment epithelial cells are a cell source for large amounts of extracellular vesicle production [34]. Our study demonstrates that corneoscleral-ring-derived extracellular vesicles, which are limbal tissue-derived, provide a valuable tissue source for producing a large quantity of extracellular vesicles within 24 h without the need for cell culture. For corneoscleral-ring-derived extracellular vesicles, donor origin, donor age, corneal endothelial density, and underlying disease were not confounding factors affecting extracellular vesicle size or concentration.

In the study, corneoscleral-ring-derived extracellular vesicles expressed transmembrane protein (CD9, CD63, CD81) and extracellular vesicle biosynthesis cytosolic protein (Alix). Among the heterogeneous e markers expressed on corneoscleral-ring-derived extracellular vesicles [35,36], CD9 and CD81 were relatively stable, and Integrin α9 was also detected, indicating the presence of LSC-derived extracellular vesicle fraction. Furthermore, Integrin α9 may serve as a useful marker for identifying or isolating LSC-derived extracellular vesicles.

In the corneoscleral-ring-derived extracellular vesicles, CD63 exhibited high heterogeneity. Moreover, the NanoFCM analysis revealed difference in CD63 expression on the Integrin α9+ and Integrin α9− extracellular vesicles. The heterogeneity of CD63 in the corneoscleral-ring-derived extracellular vesicles may due to the proportion of LSC-derived extracellular vesicles’ fraction.

Our studies also revealed donor-related heterogeneity in the expression of ABCG2, p63, Notch-1, and Integrin α9 in the corneoscleral-ring-derived extracellular vesicles, reflecting the intrinsic biological variation in extracellular vesicle composition [37]. Of particular interest is Integrin α9, a transmembrane adhesion molecule that plays a central role in cell adhesion and migration and is crucial for wound healing [38,39]. A previous study had found that the Integrin α9 was unique in limbal tissue, but not presented in mature corneal tissue [25]. Also, Integrin α9 expression was limited to certain basal cells of the limbal epithelia [28]. Willow et al. had revealed that Integrin α9 contributes to wound healing by promoting cell migration, proliferation, and proper cytoskeletal dynamics, both in vitro and in vivo [40]. In our results, corneoscleral-ring-derived extracellular vesicles contained 7–10% Integrin α9+ extracellular vesicles and exhibited greater wound healing potency compared with extracellular vesicles derived from MSCs and RPE cells. These findings suggest that Integrin α9+ extracellular vesicles may have enhanced potential to support corneal repair.

TFF is widely used for the isolation of extracellular vesicles and can effectively remove residual proteins from the conditioned medium [30]. After TFF processing, the corneoscleral-ring-derived extracellular vesicles retained their intrinsic wound healing potency. Further profiling of miRNAs and proteins in these corneoscleral-ring-derived extracellular vesicles using RNA sequencing will be important to elucidate the molecular mechanisms underlying their corneal repair function.

In this study, donors with underlying cancer or immunodeficiency did not exhibit any impact on extracellular vesicle characteristics or corneal wound repair (Figure 8 and Figure S2). As the cornea is a vessel-free tissue and the limbal region actively suppressed angiogenesis, these features may limit potential influences of systemic disease on corneoscleral-ring-derived extracellular vesicles. Our data indicated that extracellular vesicles from healthy (Limbo-Exo-18), cancer (Limbo-Exo-1, 8, 13, 20), and immunodeficiency (Lim-Exo-4) donors displayed comparable wound healing potency (Figure 8). Although we have demonstrated through several characterization techniques that the extracellular vesicle characteristics of donor tissues were not affected by cancer or immunodeficiency, using cancer- or immunodeficiency-donor-derived extracellular vesicles for treating corneal disorders remains uncertain since disease-related alterations in the donor’s ocular microenvironment might subtly affect extracellular vesicle function. More experiences are warranted.

## 5. Conclusions

In conclusion, we characterized human corneoscleral-ring-derived extracellular vesicles and identified a fraction of extracellular vesicles with Integrin a9 expression regarding their origin of LSCs. The superior wound healing potency of human corneoscleral-ring-derived extracellular vesicles highlights their promise as a therapeutic candidate for ocular surface repairment.

## Figures and Tables

**Table 1:** Baseline information of cornea donor.

Limbo-Exo No.	Origin	Age	Sex	Primary Cause of Death	Corneal Endothelial Density (Cells/mm^2^)	Duration ^a^	Extracellular Vesicles Concentration (Particles/mL)	Extracellular Vesicles Peak Size (Diameter/nm)	PDI	Span
1	US	64	unknown	liver CA	2801	14	113	2 × 10^11^	0.37	1.40
2	US	65	F	CHF	3006	18	101	3× 10^11^	0.35	1.60
3	US	61	M	CHF	2942	19	103.1	4 × 10^11^	0.34	1.47
4	US	68	unknown	SAH	2890	18	104.1	4 × 10^11^	0.24	1.37
5	Taiwan	44	M	SAH	2710	17	95.5	6.1 × 10^11^	0.19	1.37
6	Taiwan	7	F	Thrombocytopenia	3226	13	114.8	4.8 × 10^10^	0.18	1.16
7	Taiwan	40	M	EDH	2747	16	100.5	3.2 × 10^10^	0.22	1.23
8	Taiwan	59	F	cancer	2169	15	90.7	9.0 × 10^10^	0.27	1.87
9	US	61	M	CHF	2855	17	103.2	3.8 × 10^11^	0.27	1.46
10	US	68	unknown	SAH	2825	18	106	4.6 × 10^11^	0.27	1.41
11	US	73	unknown	lung CA	2358	14	92.9	2.4 × 10^11^	0.25	1.44
12	US	78	F	dyspnea	2841	15	98.6	6.4 × 10^10^	0.30	1.53
13	US	78	F	dyspnea	2865	19	101.1	7 × 10^10^	0.59	1.76
14	Taiwan	44	M	SAH	2688	17	109.0	1.1 × 10^11^	0.23	1.33
15	Taiwan	40	M	cancer	2833	17	100.9	1.20 × 10^11^	0.21	1.53
16	Taiwan	59	F	cancer	2632	15	126.9	1.0 × 10^10^	0.41	2.06
17	Taiwan	18	M	SAH	3448	18	110.2	2.80 × 10^11^	0.25	1.29
18	Taiwan	18	M	SAH	3425	18	105.8	3.10 × 10^11^	0.31	1.42
19	Taiwan	39	M	spine tumor	2841	18	102.5	2.70 × 10^11^	0.20	1.30
20	Taiwan	39	M	spine tumor	2941	18	101.5	2.90 × 10^11^	0.23	1.29
21	Taiwan	49	M	amyotrophic lateral sclerosis	2639	13	94	2.60 × 10^11^	0.21	1.35
22	Taiwan	44	M	respiratory failure	2740	12	71.9	3.00 × 10^11^	0.43	1.43
23	Taiwan	41	M	respiratory failure	2809	17	87.7	3.00 × 10^11^	0.27	1.56
24	Taiwan	52	M	respiratory failure	2646	20	74.2	4.00 × 10^11^	0.29	1.74
25	Taiwan	52	M	respiratory failure	2646	20	81.6	4.40 × 10^11^	0.25	1.19
26	Taiwan	55	M	cancer	2801	14	99.1	3.00 × 10^11^	0.26	1.39
27	Taiwan	36	M	brain tumor	3484	15	98.6	3.40 × 10^11^	0.17	1.28
28	Taiwan	49	F	breast cancer	2639	15	113.1	8.10 × 10^11^	0.23	1.25
29	Taiwan	43	M	hypoxia	2882	20	97.1	2.20 × 10^11^	0.32	0.97
30	Taiwan	43	M	hypoxia	3012	20	107.9	2.30 × 10^11^	0.32	1.13
31	US	65	unknown	NSTEMI	2347	13	101.3	2 × 10^11^	0.13	0.89
32	US	57	unknown	COPD	2513	19	100.3	1.2 × 10^11^	0.14	1.08
33	US	75	unknown	CHF	2404	16	75.3	1.5 × 10^11^	0.15	1.11
34	US	48	unknown	pneumonia	2611	17	106.1	1.8 × 10^11^	0.22	1.21
35	US	46	unknown	pulmonary embolism	2506	17	104.2	2.6 × 10^11^	0.23	1.31
36	US	65	unknown	ESRD	2584	17	101.8	1.7 × 10^11^	0.19	1.34
37	US	46	unknown	pulmonary embolism	2404	17	104.8	2.5 × 10^11^	0.24	1.40
38	US	66	unknown	CHF	2833	19	71.3	2.4 × 10^11^	0.25	1.11
39	US	43	M	ICH	2747	19	126	1.9 × 10^11^	0.17	1.09
40	US	58	M	pulmonary fibrosis	3125	18	70.4	2.2 × 10^11^	0.21	1.00
41	US	58	M	pulmonary fibrosis	3514	18	105.8	2.8 × 10^11^	0.31	1.64
42	US	54	unknown	trauma	3012	19	106.2	3.1 × 10^11^	0.30	1.43
Mean		51.62 ± 15.56			2808.12 ± 304.20	16.88 ± 2.15	99.52 ± 13.00	2.58 × 10^11^ ± 1.53 × 10^11^	0.26 ± 0.08	1.36 ± 0.24

^a^: from date of death to extracellular vesicles extraction; CA = carcinoma; CHF = chronic heart failure; SAH = subarachnoid hemorrhage; PDI = polydispersity index; EDH = epidural hemorrhage; NSTEMI = non ST elevation myocardial infraction; COPD = chronic obstructive pulmonary disease; ESRD = end-stage renal disease; ICH = intracerebral hemorrhage.

## Data Availability

The data presented in this study are available upon request from the corresponding authors.

## Supplementary Materials

The following supporting information can be downloaded at: https://www.mdpi.com/article/10.3390/life15111780/s1, Figure S1: Extracellular vesicle and donor characteristics by donor age group; Figure S2: Extracellular vesicle characteristics by donor medical history.

## Abbreviations

The following abbreviations are used in this manuscript:

ABCG2	ATP-binding cassette subfamily G member 2
AMD	Age-related macular degeneration
DPBS	Dulbecco’s phosphate-buffered saline
DLS	Dynamic light scattering
dUC	Differential ultracentrifugation
ECD	Endothelial cell density
HCET	Human Corneal Epithelial-transformed cell
LSC	Limbal stem cell
MEM α	Minimum essential medium α
MSC	Mesenchymal stem cell
MVBs	Multivesicular Bodies
NanoFCM	Nanoparticle flow cytometry
NTA	Nanoparticle tracking analysis
PBS	Phosphate-buffered saline
RPE	Retinal pigment epithelia
TEM	Transmission electron microscopy
TFF	Tangential flow filtration
UA	Uranyl acetate
