# Supplementary material for: Isolation and Characterization of Integrin α9 Positive Extracellular Vesicles Derived from Human Corneoscleral Rings
Source: Life (Basel). 2025 Nov 20;15(11):1780. doi: 10.3390/life15111780 (PMC12654178; doi:10.3390/life15111780)
Supplement: Supplementary file 1 [file life-15-01780-s001.zip › life-3947889-supplementary.pdf]

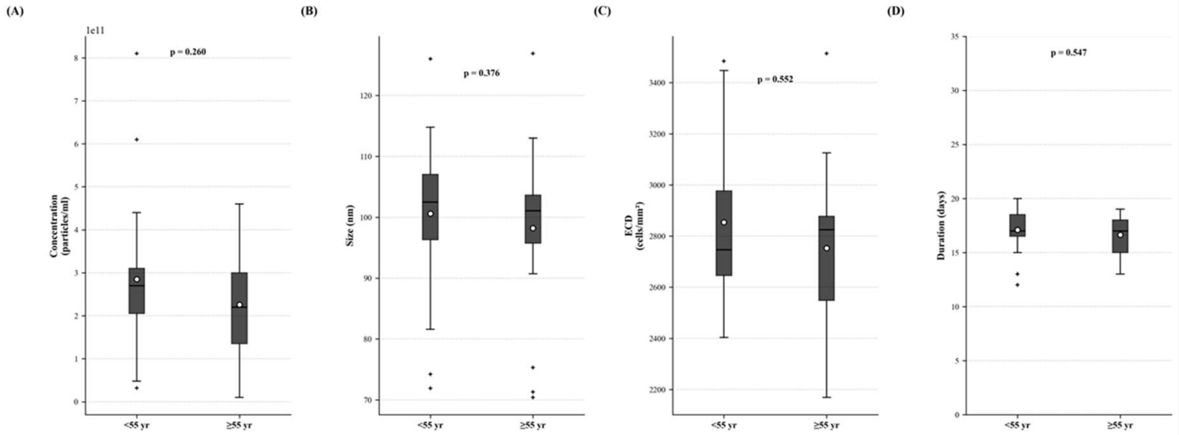

Figure S1. Extracellular vesicle and donor characteristics by donor age group.

Supplementary Figure S1. Legend. Extracellular vesicle and donor characteristics by donor age group. Comparison of extracellular vesicle characteristics by donor age. (A) Concentration ($\times 10^{11}$ particles/ml), (B) size (nm), (C) corneal endothelial cell density (ECD, cells/mm^2), and (D) duration from death to extracellular vesicle extraction (days) for donors <55 years vs ≥55 years. Box plots show median, quartiles, and 1.5× IQR whiskers; + = outliers; ○ = means. P-values from Mann-Whitney U test.

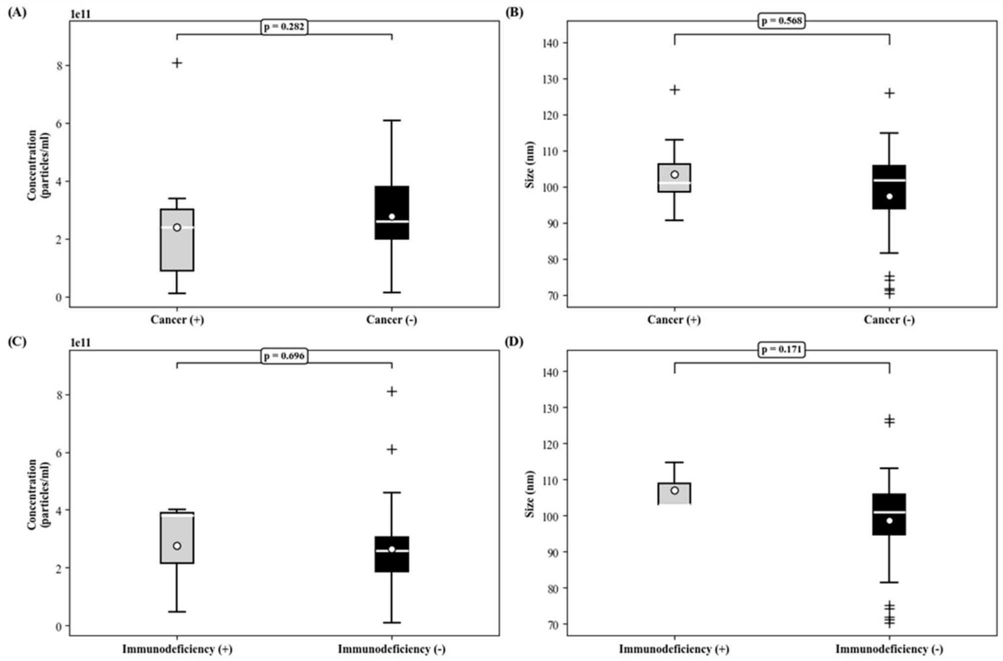

Figure S2. Extracellular vesicle characteristics by donor medical history.

Supplementary Figure S2. Legend. Extracellular vesicle characteristics by donor medical history. Comparison of extracellular vesicle concentration (×10¹1 particles/ml) and size (nm) between donors with versus without (A,B) cancer history and (C,D) immunodeficiency history. Box plots show median, quartiles, and 1.5× IQR whiskers; + = outliers; ○ = means. Statistical comparisons by Mann-Whitney U test.
